# A case series of intestinal adenomatous polyposis of unidentified etiology; a late effect of irradiation?

**DOI:** 10.1186/s12885-016-2880-2

**Published:** 2016-11-08

**Authors:** Lisanne Sara Rigter, Frank G. J. Kallenberg, Barbara Bastiaansen, Theo A. M. van Os, Floor E. van Leeuwen, Monique Esther van Leerdam, Evelien Dekker

**Affiliations:** 1 Department of Gastroenterology, The Netherlands Cancer Institute, Amsterdam, The Netherlands; 2 Department of Gastroenterology and Hepatology, Academic Medical Center, Meibergdreef 9, 1105 AZ Amsterdam, The Netherlands; 3 Department of Clinical Genetics, Academic Medical Center, Amsterdam, The Netherlands; 4 Division of Epidemiology, The Netherlands Cancer Institute, Amsterdam, The Netherlands

**Keywords:** Cancer survivor, Intestinal polyposis, Gastrointestinal malignancies, Radiotherapy

## Abstract

**Background:**

In a large number of patients with multiple gastrointestinal adenomatous polyps, no causal germline mutation can be found. Non-genetic factors may contribute to the development of adenomatous polyps in these unexplained polyposis patients.

In the development of gastrointestinal cancer, prior exposure to abdominal radiotherapy has been identified as such a factor, as it increases the gastrointestinal cancer risk in cancer survivors. A relationship of radiotherapy with intestinal polyposis, however, has not yet been described. Despite the increased cancer risk, these cancer survivors do not receive gastrointestinal screening recommendations. This case series describes three patients with adenomatous polyposis after abdominal radiotherapy.

**Case presentation:**

Patient 1 was diagnosed with testicular cancer at the age of 31 and was treated with hemicastration, radiotherapy and chemotherapy. Thirty-nine years later, he was diagnosed with more than 30 colonic adenomas. Additionally, gastroduodenoscopy revealed a well-differentiated adenocarcinoma in the antrum of the stomach.

Patient 2 was diagnosed with a nephroblastoma at the age of 10, which was resected and treated with radiotherapy and chemotherapy. At age 36, a rectal adenocarcinoma was diagnosed and treated by radiotherapy and a total mesorectal excision. During 11 years of surveillance endoscopies, 21 colonic adenomas and three duodenal adenomas were detected.

Patient 3 was diagnosed with Hodgkin lymphoma at the age of 20 and treated with radiotherapy, followed by chemotherapy for a recurrence 3 years later. At age 62, a subtotal colectomy was performed because of colonic polyposis: 36 adenomas were detected. During screening gastro-duodenoscopy, three duodenal adenomas were detected.

In all three patients, germline analysis did not reveal a mutation in the *APC* and *MYH* genes*.* The gastric and rectal cancer were both microsatellite stable.

**Conclusion:**

This report describes three patients with adenomatous polyposis, of which two developed a gastrointestinal cancer. The polyposis was not explained by a germline mutation in *APC* or *MYH* and all patients received abdominal radiotherapy. Although an etiologic role has not been established, an association between radiotherapy and intestinal adenomatous polyposis and the subsequent development of cancer seems very likely in our patients.

## Background

Patients with classical familial adenomatous polyposis (FAP) present with over 100 adenomatous colonic polyps, whereas patients with attenuated FAP (AFAP) have approximately 10−100 adenomatous polyps. These syndromes are often explained by a genetic mutation in the adenomatous polyposis coli (*APC*) gene. A small number of cases of polyposis are caused by other germline mutations, in genes such as MutY homolog (*MYH*), DNA polymerase ɛ *(POLE)* and δ *(POLD1)* [1–3]. However, in a significant number of cases no mutation can be detected: 10−30 % for patients with over 100 adenomas and up to 90 % for patients with approximately 10−90 adenomas [4–6]. These adenomatous polyposis syndromes without a confirmed germline mutation can be referred to as ‘clinical FAP’ and ‘adenomatous polyposis of unknown origin’ respectively [7].

Several non-genetic factors have been associated with an increased risk of developing multiple intestinal polyps and cancer, such as high age, smoking and high body mass index [8, 9]. There is evidence in the literature that prior cancer treatment, e.g. abdominal radiotherapy and several chemotherapeutics (especially alkylating agents), also increases the risk for the development of gastrointestinal cancer [10–16]. Many cancer survivors have an increased risk of developing gastrointestinal malignancies, including survivors of Hodgkin lymphoma, testicular cancer and nephroblastoma [12, 14–22]. The reported risks of these cancer survivors range from 2- to 13-fold for gastric cancer, 11-fold for cancer in the small bowel and 2- to 7-fold for colorectal cancer.

Although it is likely that these cancers arise from precancerous lesions, an association between radiotherapy and intestinal polyposis has not yet been established and the prevalence of intestinal polyps and the pattern of radiation-induced carcinogenesis is unknown. Because of the lack of this knowledge, these cancer survivors do not receive screening or surveillance recommendations. This case series describes three Caucasian patients with intestinal adenomatous polyposis after abdominal radiotherapy for a prior malignancy.

## Case presentation

### Case 1

A 70-year-old asymptomatic man underwent a colonoscopy after a positive fecal immunochemical test that was performed within the national colorectal cancer (CRC) screening program. The patients’ medical history consisted of a malignant teratoma with intermediate differentiation of the right testis and a positive lymphography at the age of 31 years. He was treated with hemicastration and external beam radiotherapy (8 megavolt (MV) photons, opposed fields, one field per day) on both lungs (20 Gray (Gy)), all retroperitoneal lymph nodes (40 Gy in 24 fractions) and a surdosage on para-aortic lymph nodes (10 Gy in 5 fractions). Nine months after diagnosis, jugular lymph node and lung metastases were detected and treated with additional chemotherapy (vincristine and actinomycin, followed by vinblastine injections, which were continued for 2 years). In addition, the patient suffered from two myocardial infarctions at the age of 51. The patient was a non-smoker, drank 5 units of alcohol per week and his body mass index (BMI) was 26.1.

**Fig. 1:**
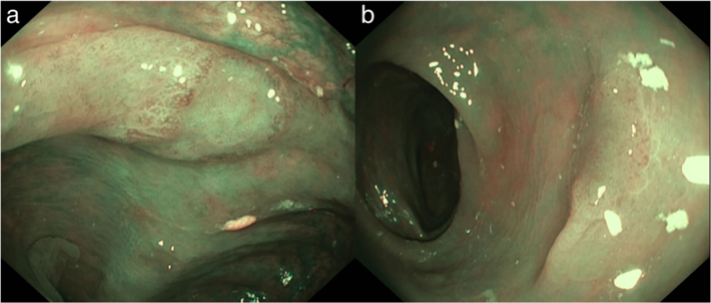
Endoscopic images of patient 1. **a**: Flat adenomatous lesion, non-granular type (Kudo 3 s), approximately 10 mm, descending colon (with narrow-band imaging). **b**: Flat adenomatous lesion, non-granular type (Kudo 3 s), approximately 10 mm, transverse colon (with narrow-band imaging)

At colonoscopy, more than 30 adenomatous polyps with a maximum size up to 20 mm were detected, mostly located in the right colon (Fig. 1). Several of these polyps were removed and histopathology demonstrated tubular and tubulovillous adenomas with low-grade and high-grade dysplasia. As these large flat colonic polyps were numerous and difficult to detect and delineate, surveillance was considered suboptimal and a subtotal colectomy with ileosigmoidal anastomosis was advised. However, as the patient declined surgery, continuous colonoscopy surveillance is scheduled.

As polyposis patients also have an increased risk for duodenal polyps and cancer, a screening gastro-duodenoscopy was performed. No duodenal polyps were identified. However, an erosive pangastritis was seen with a poorly circumscribed, slightly elevated erythematous region of approximately 4 cm, located in the greater curvature of the antrum. This region was biopsied and histopathology demonstrated adenomatous tissue with high-grade dysplasia. Endoscopic submucosal dissection of the gastric lesion was performed, and histopathology demonstrated a well-differentiated adenocarcinoma with invasion into the muscularis mucosae (pT1m3NxMx) in the background of low- and high-grade dysplasia. No additional oncologic treatment was indicated as this T1 lesion was radically resected. During this procedure, a second irregularly shaped and slightly elevated lesion of 3 mm was detected in the stomach and removed, which appeared an adenoma with low-grade dysplasia.

For further analysis of the intestinal polyposis, the patient was referred to a clinical geneticist. His father and father’s brother were both diagnosed with colorectal cancer at the age of 65 and 86 years, respectively. No germline mutation was detected in the *CDH1*, *APC* or *MYH* genes and the gastric adenocarcinoma was microsatellite stable with normal expression of the mismatch repair genes (*MLH1, MSH2, MSH6, PMS2*). A multigene custom panel containing polyposis-associated genes did not reveal any additional germline mutations in *ACVRL1, AXIN2, BMPR1A, ENG, EPCAM, GREM1, MLH1, MSH2, MSH6, NTHL1, PMS2, POLD1, POLE, PTEN, SMAD4, STK11, TSC1 and TSC2* (SeqCap Ez choice, Nimblegen).

### Case 2

This case describes a woman who was diagnosed with a nephroblastoma of the right kidney at the age of 10. The tumor was treated with neoadjuvant radiotherapy (20 Gy on the abdomen and a surdosage of 10 Gy on the tumor, in 19 fractions, external beam radiotherapy, ≤9 MV photons), nephrectomy, and six courses of dactinomycin and vincristine. At 18 years, an osteosarcoma of the sixth rib was resected.

At age 36, she presented with bloody diarrhea. Colonoscopy revealed a rectal adenocarcinoma, which was treated by 5x5 Gy radiotherapy and a total mesorectal excision. The moderately differentiated adenocarcinoma, ypT2N0M0, was radically resected. She was a non-smoker, who did not consume alcohol, with a BMI of 25.6.

**Fig. 2:**
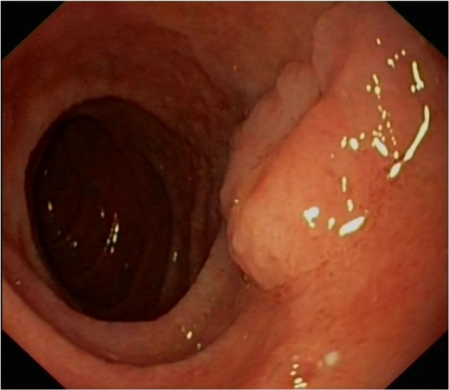
Endoscopic images of patient 2. Sessile adenomatous lesion, approximately 20 mm, duodenum

During 11 years of follow up, surveillance colonoscopies revealed 21 adenomas, which were removed (tubular adenomas with low-grade dysplasia and one tubular adenoma with high-grade dysplasia). A screening gastroduodenoscopy was performed, resulting in the detection of one 3 mm tubular adenoma with low-grade dysplasia in the descending duodenum. Random sampling of the gastric mucosa did not reveal any metaplasia or dysplasia. During annual gastroduodenal surveillance, two additional tubulovillous adenomas with low-grade dysplasia (max. diameter 15 mm) were detected and resected in the duodenum (Fig. 2).

Because of the polyposis and the young onset of CRC, the patient was referred to a clinical geneticist. The only family member with cancer was her father’s brother who was diagnosed with renal cancer at unknown age. The patient was tested for germline mutations in the *APC*, *MYH and TP53* genes, revealing no mutation*.* In addition, the rectal adenocarcinoma appeared microsatellite stable with normal expression of the mismatch repair genes (*MLH1, MSH2, MSH6, PMS2*).

### Case 3

Our third patient was diagnosed with stage IIA Hodgkin lymphoma at the age 20 years. He was treated with irradiation of the neck, axillary and mediastinal nodes (35 Gy). After 3 years, he developed a recurrence and was treated with additional para-aortic and iliac radiotherapy (40 Gy in 22 fractions, external beam radiotherapy, 8 MV photons, opposed fields, one field per day). A second recurrence was treated with radiotherapy on the left axilla (40 Gy) and 12 courses of MOPP (mechlorethamine, vincristine, procarbazine, prednisone). At the age of 62, he presented with epigastric pain. The patient consumed alcohol socially and smoked 8–10 cigarettes per day. His BMI was 19.0.

A gastro-duodenoscopy was performed, which demonstrated no gastric abnormalities. In the duodenum, however, a sessile polyp of 8 mm was detected and endoscopically removed. Histopathology demonstrated a tubular adenoma with low-grade dysplasia. During follow-up, two small tubular adenomas with low-grade dysplasia of the duodenum were detected, of which one was removed. Surveillance gastroduodenoscopies are scheduled.

**Fig. 3:**
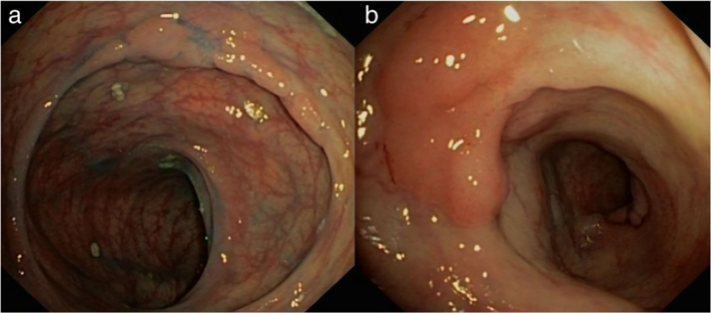
Endoscopic images of patient 3. **a**: Lateral spreading flat adenomatous lesion on top of a colonic fold, non-granular type (Kudo 3 s), approximately 20−25 mm, descending colon. **b**: Flat elevated adenomatous lesion, non-granular type (Kudo 3 s), approximately 15−20 mm, descending colon

Because of the known association between duodenal and colonic adenomas, a colonoscopy was performed. Approximately 30 adenomas were detected throughout the colon (Fig. 3) for which a prophylactic subtotal colectomy with ileorectal anastomosis was performed. In total, this patient had a total of 36 adenomas with low-grade and high-grade dysplasia and several hyperplastic polyps (including all endoscopically resected polyps and polyps in the surgical specimen).

The family history revealed that one second-degree relative (his mother’s brother) was diagnosed with CRC at the age of 87 years. The clinical geneticist did not detect a germline mutation in the *APC* or *MYH* gene.

## Discussion

This report described three patients with intestinal adenomatous polyposis that could not be explained by a germline mutation in the *APC* or *MYH* gene, the most common mutations for this condition [23]. All three patients developed intestinal polyposis after receiving abdominal radiotherapy for different malignancies, with a latency of 26–42 years. The radiotherapy fields of these cases largely overlapped with the presence of adenomas. The development of intestinal cancer most likely occurs within an irradiated segment [10]. Abdominal radiotherapy fields deliver the highest exposure to the transverse colon (para-aortic field) and the cecum and rectosigmoid (iliac fields) [24]. In the stomach, the antrum is the site that is exposed most extensively (para-aortic field) [14].

As radiotherapy is known to accelerate gastrointestinal carcinogenesis in mouse models and increases gastrointestinal cancer risk in humans, a causal role for radiotherapy in the development of intestinal adenomatous polyposis seems plausible [10, 11, 25, 26]. These two arguments also apply for the potential causal role for alkylating agents [10, 11, 25, 27, 28]. One of our patients also received alkylating agents, including procarbazine, which may have attributed to the development of adenomatous polyposis.

When assessing the cause of adenomatous polyposis, multiple factors should be addressed. Firstly, in a large proportion of patients with less than 100 adenomas, genetic analysis of the germline mutations *APC* and *MYH* is not conclusive, which results in a diagnosis of adenomatous polyposis of unknown origin [6, 7]. The development of adenomatous polyposis could theoretically also be explained by unclassified or unrecognized mutations in polyposis-associated genes, mutations in unidentified polyposis-associated genes or mosaicism [23, 29]. In addition, the recently discovered mutations in genes such as *POLE* and *POLD1* have not (yet) been evaluated in two of three cases [3]. Although acquired genetic aberrations have never been discovered in the etiology of polyposis, epidemiologic studies have also described non-inherited risk factors for polyposis, such as age, smoking and body mass index [8, 9]. We believe that the contribution of these factors, however, is very limited.

Only a few case reports on radiation-induced CRC have been published. They confirm that the development of normal mucosa into radiation-induced gastrointestinal adenocarcinomas is poorly investigated and hypothesize that cancer may arise from flat adenomas and/or an inflammatory background [30–32]. A recent report showed a cumulative risk of 16.4 % for developing a subsequent primary cancer beyond 30 years after treatment for Wilms tumor; 41 % of these excess cancers, all treated with abdominal radiotherapy, were attributable to digestive cancer [16]. Up to date, only one case report on radiotherapy- and chemotherapy-associated intestinal adenomatous polyposis has been published [33]. This report is limited to five childhood cancer survivors who developed adenomatous polyposis, but did not develop malignancies at follow up. This may be explained by the relative low mean age of endoscopy at 24.8 years (range 13−35) which is lower than the age of colonoscopy in our cases. This raises the possibility that the development of cancer was prevented by early detection and removal of polyps in those patients. The authors conclude that endoscopic surveillance may be indicated [33]. In our patients, earlier surveillance potentially may have prevented both the rectal and gastric cancer.

Although the significance of our case report is limited by the number and heterogeneity of cases, it does raise the question whether endoscopic surveillance is indicated to prevent cancer in patients who received abdominal radiotherapy. The increased risk of gastrointestinal malignancies seems to rise at 10 years after the primary cancer treatment [10, 13], and the risk remains increased after a follow-up period of over 30 years [12, 16–18, 34, 35], When considering these risks, one should take into account the fact that in more recent years a lowering of therapeutic radiation exposure has resulted in a decline in late-mortality among childhood cancer survivors over the last decades [35].

In our opinion, the long-term increased risk for gastrointestinal cancer in cancer survivors underscores the need for a personalized surveillance recommendation in those patients. For childhood cancer survivors who received 30 Gy or more abdominal irradiation, the American Children’s Oncology Group recommends colonoscopy surveillance every 5 years beginning at 35 years [36]. The Dutch cancer survivor guidelines do not include surveillance for gastrointestinal cancer, because important criteria for surveillance are not met, including information on the pathogenesis of radiation- and chemotherapy-induced malignancies and on risk stratification in subgroups of cancer survivors. Currently, a prospective cohort study of Hodgkin lymphoma survivors who received abdominal radiotherapy and/or chemotherapy at increased CRC risk is ongoing in the Netherlands, evaluating the efficacy of a CRC screening program in these patients. (Dutch Trial Registry NTR4961).

## Conclusions

In conclusion, these three patients with intestinal adenomatous polyposis of which two developed cancer, demonstrate the potential causal role of radiotherapy. For cancer survivors who were treated with abdominal radiotherapy, a delay in the diagnosis of gastrointestinal cancer may be prevented by awareness of the increased risk. Further research on the etiology of adenomatous polyposis and gastrointestinal cancer in those cancer survivors and on optimal surveillance and prevention methods is urgently needed.

## Abbreviations

AFAP: Attenuated familial adenomatous polyposis
*APC*: Adenomatous polyposis coli
BMI: Body mass index
CRC: Colorectal cancer
FAP: Familial adenomatous polyposis
Gy: Gray
MV: Megavolt
MOPP: Mechlorethamine, vincristine, procarbazine, prednisone
*MYH*: MutY homolog
*POLD1*: DNA polymerase δ
*POLE*: DNA polymerase ɛ
